# Recursive least squares background prediction of univariate syndromic surveillance data

**DOI:** 10.1186/1472-6947-9-4

**Published:** 2009-01-16

**Authors:** Amir-Homayoon Najmi, Howard Burkom

**Affiliations:** 1 National Security Technology Department, The Johns Hopkins University Applied Physics Laboratory, Laurel, MD 20723-6099, USA

## Abstract

**Background:**

Surveillance of univariate syndromic data as a means of potential indicator of developing public health conditions has been used extensively. This paper aims to improve the performance of detecting outbreaks by using a background forecasting algorithm based on the adaptive recursive least squares method combined with a novel treatment of the Day of the Week effect.

**Methods:**

Previous work by the first author has suggested that univariate recursive least squares analysis of syndromic data can be used to characterize the background upon which a prediction and detection component of a biosurvellance system may be built. An adaptive implementation is used to deal with data non-stationarity. In this paper we develop and implement the RLS method for background estimation of univariate data. The distinctly dissimilar distribution of data for different days of the week, however, can affect filter implementations adversely, and so a novel procedure based on linear transformations of the sorted values of the daily counts is introduced. Seven-days ahead daily predicted counts are used as background estimates. A signal injection procedure is used to examine the integrated algorithm's ability to detect synthetic anomalies in real syndromic time series. We compare the method to a baseline CDC forecasting algorithm known as the W2 method.

**Results:**

We present detection results in the form of Receiver Operating Characteristic curve values for four different injected signal to noise ratios using 16 sets of syndromic data. We find improvements in the false alarm probabilities when compared to the baseline W2 background forecasts.

**Conclusion:**

The current paper introduces a prediction approach for city-level biosurveillance data streams such as time series of outpatient clinic visits and sales of over-the-counter remedies. This approach uses RLS filters modified by a correction for the weekly patterns often seen in these data series, and a threshold detection algorithm from the residuals of the RLS forecasts. We compare the detection performance of this algorithm to the W2 method recently implemented at CDC. The modified RLS method gives consistently better sensitivity at multiple background alert rates, and we recommend that it should be considered for routine application in bio-surveillance systems.

## Background

Timely detection of an outbreak is a major goal of surveillance of public health data. Many techniques in the last several years have been developed to address anomaly detection in univariate time series. For instance, the CDC's current methods for time series aberration detection are based on Xbar and CUSUM control charts [1]. Others have used Auto-Regressive Integrated Moving Average (ARIMA) models [2], regression based models [3], and control charts based on Holt and Winters forecasting [5] with generalized exponential smoothing. We have selected the more recent CDC method known as the W2. The W2 method uses a fixed number of days (usually a multiple of 7) as a sliding window and tracks the mean value of the weekdays and weekends separately, with a 2 day buffer between the baseline and test data. [6].

Multistream (multivariate) anomaly detection has also received some attention in the hope that a major outbreak could have early indications in some streams. For example, over the counter medication sales as a potential early indicator of developing public health conditions, in particular in cases of interest to biosurvellance, has been suggested in the literature [7]. In such cases, and using multi stream data that have been shown to have correlations with the syndromic data of interest, a several-steps ahead forecast of the syndromic data is made using all available streams, and in most instances a threshold detector is used to indicate an outbreak. The reason for forecasting several steps ahead is that a next-step predictior has the risk of training on early signal effects, so that later and stronger signal effects will be within expected limits.

In a previous publication [8] we presented a prediction and detection method based on an adaptive recursive least squares filter applied to time series derived from emergency department (ED) and OTC data. We compared those predictions and detections with similar predictions that use the same method applied to the clinical data alone without referencing any other data streams. We found that when we used the past values of the syndromic time series alone to predict its future values, the prediction errors were consistently at least as low (in the sense of minmum squared error) as predictions using the reference (OTC) time series. Consequently we suggested that the univariate method can be a useful background predictor, which is the subject of the present paper. The method could be summarized as follows. A single syndromic time series is used for many-steps ahead predictions of itself. These predictions are used as estimates of the "normal" background behaviour of the syndromic data. A comparison between the estimated background value and the actual daily count (on a given day) is then used to declare the presence or the absence of an anomaly on that day.

The finite impulse response implementation of the background predictor is, however, adversely affected by several issues. Many syndromic time series have low counts, and in these cases any potential advantages over simpler methods are lost. So we limit our study to those syndromic series that have mean and median daily counts of more than 100. Seasonal fluctuations are sufficiently long in period that our adaptive methods are quite capable of handling. However, a strong Day of the Week effect (DOW) has too short a period, and we illustrate the DOW problem using the respiratory-1 syndromic data from military outpatient clinics of a major metropolitan area in the United States. We use a definition of Respiratory illness that can be defined as acute infection of the upper and/or lower respiratory tract, excluding chronic conditions such as chronic bronchitis, asthma, and sinusitis [4].

**Figure 1:**
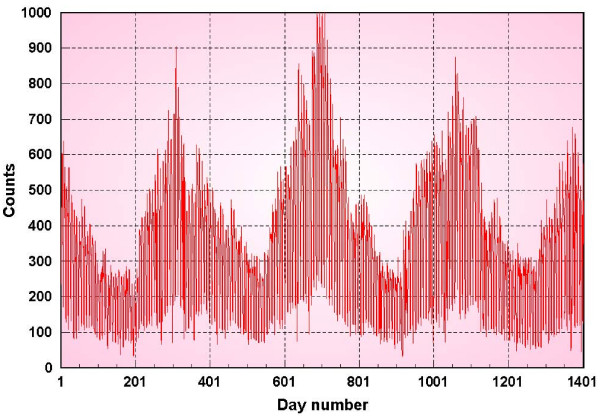
**Respiratory 1 syndromic data**.

**Figure 2:**
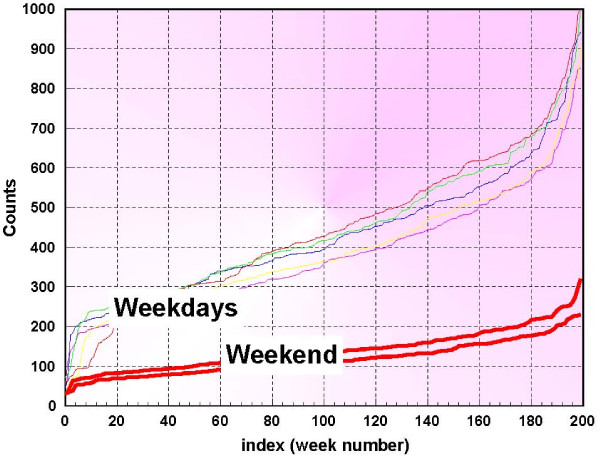
**Respiratory 1 sorted syndromic data for different days of the week**.

Figure 1 shows the daily counts for a 3-year period 1994–1997. The DOW effect is seen as the extreme drop in daily counts for Saturdays and Sundays. Another illustration of this effect is shown in figure 2 which depicts the sorted values of the daily counts for each day of the week.

Figure 2 is constructed in the following way. Let *d*_*j *_[*n*] denote the daily counts for day number *j*, *j *= 1,..., 7. With this notation, the 1^*st *^week's data would be *d*_1 _[1], *d*_2 _[1],..., *d*_7 _[1]. Now we sort each {*d*_*j *_[*n*], *n *= 1, 2,...} by their values, which are now depicted in figure 2. Note that a cumulative distribution function can be computed using these curves. For instance, if the number of days that daily counts of a given day number have not exceeded a given level, i.e. days for which *d*_*j *_≤ *D*_*j*_, is denoted by $n_{D_{j}}$, then $p\{d_{j}\leq D_{j}\}=\frac{n_{D_{j}}}{N}$ where *N *is the the number of occurences of day of week *j *in the baseline. Denoting the syndromic time series on day number *n *by *y*[*n*] the k-step ahead prediction problem is to estimate future values of the clinical data $\hat{y}$[*n *+*k*], *k *> 0, using past days' values of the same clinical data. In the next section, we apply the individual day-of-week distribution functions above to enable recursive least squares (RLS) k-step ahead predictions for this estimate. The RLS method too is described in the next section [see additional file 1].

## Methods

As indicated in the previous section we deal exclusively with syndromic time series whose mean and median daily counts are greater than 100 [e.g., see Table 1]. The first step of our analysis is to normalize the data for the DOW effect. We use the first 30% of the time series to begin the process.

**Table 1:** Time series data from military clinics – means and standard deviations

Data Descriptor	Mean	SDev
**Resp 1**	335	196

**Resp-City A-Military Prescriptions**	829	449

**Resp-City B-Civilian Office Visit**	682	277

**Resp-City C-Civilian Office Visit**	448	239

**Resp-City A-Civilian Office Visit**	393	242

**GI-City A-Military Prescriptions**	373	167

**Resp-City C-Military Prescriptions**	368	208

**Resp-City D-Military Prescriptions**	359	225

**Resp-City A-Military Outpatient Visit**	352	177

**Resp-City E-Civilian Office Visit**	250	196

**Resp-City C-Military Outpatient Visit**	247	131

**GI-City D-Military Prescriptions**	247	126

**Resp-City D-Civilian Office Visit**	227	138

**Resp-City E-Military Prescriptions**	215	122

**GI-City C-Military Prescriptions**	214	110

**Resp-City B-Military Prescriptions**	186	90

**Figure 3:**
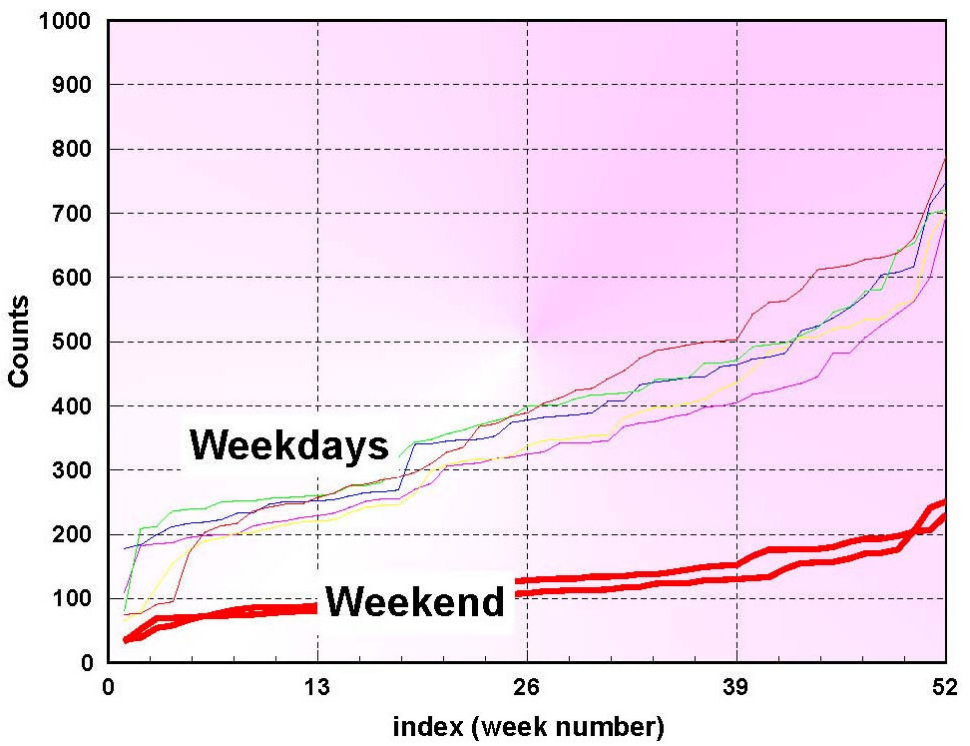
**First year respiratory 1 sorted syndromic data for different days of the week**.

**Figure 4:**
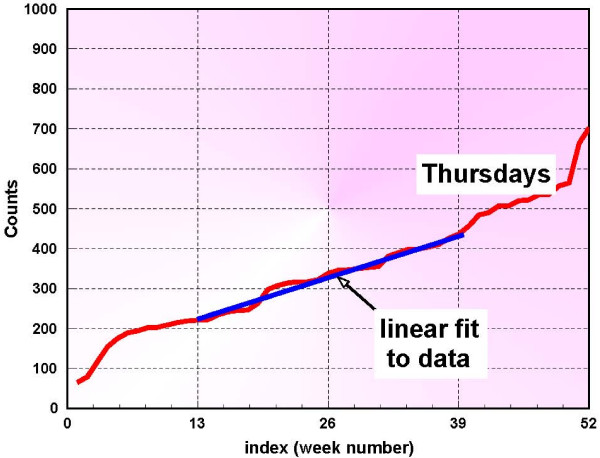
**Correction method applied to Thursdays [1 year of data]**.

The normalization method is based on the sorted data values shown in figure 2, and it is adaptive, in the sense that at each step new data enters the baseline and only the counts that are available are actually used. Figure 3 shows only the first 365 days of the data used in this study. The middle 50%, is chosen and fit with a straight line. Figure 4 is an example of this procedure, depicted for Thursdays. This process is performed for each day of the week and so 7 linear segments are found and stored. A median line is then determined by the respective medians of the 7 start points and the 7 end points. If the 7 lines are denoted by *l*_*j *_[*m*], and the median line is denoted by *L*[*m*], then at each index *m*, a set of ratios are derived from

(1) $$t_{jL}[m]=\frac{L[m]}{l_{j}[m]}$$

and applied to the sorted data values to obtain the normalized data with the day of the week effect removed:

(2) $$d_{j}^{c}[m]=t_{jL}[m]d_{j}[m].$$

**Figure 5:**
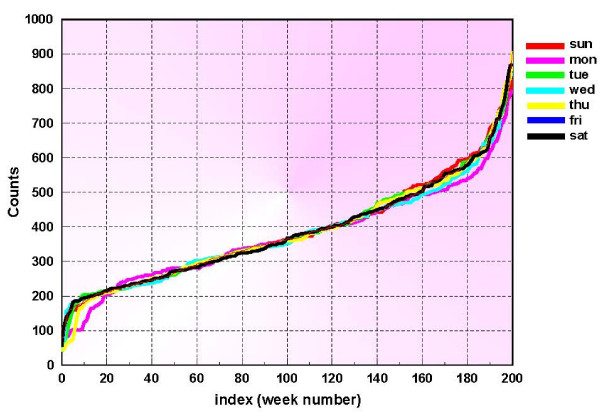
**Corrected respiratory 1 data prior to resorting [3 years of data]**.

**Figure 6:**
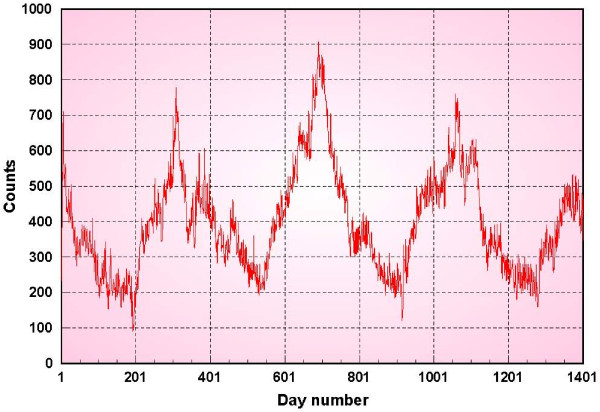
**Corrected respiratory 1 data resorted to original dates [3 years of data]**.

Normalized data $d_{j}^{c}$[*m*] are then sorted back to the original day index. Figure 5 shows the normalized data, for separate days of the week, for the entire 3 years prior to resorting to the original dates. Figure 6 shows the recombined data that have now been normalized for the day of the week effect and resorted to original dates.

The process described above is performed on successive days and the output is used to make background predictions based on the recursive least squares method that we describe now. Denoting the clinical data time series on day number *n *by *y*[*n*] the N-step ahead prediction problem is to estimate future values of the clinical data $\hat{y}$[*n *+*N*], *N *> 0, using a linear prediction equation

(3) $$\begin{array}{cc} \hat{y}[n+N]=\sum_{m=0}^{M-1}h[m]y[n-m], & N>0 \end{array}$$

and the recursive least squares method to compute the filter coefficients adaptively. The filter coefficients *h*[*m*] at day *n *are chosen by minimizing the following weighted sum of error terms (the performance index),

(4) $$\varepsilon_{n}=\sum_{k=0}^{n}\lambda^{n-k}|y_{k+N}-{\hat{y}}_{k+N}{|}^{2}$$

includes a forgetting factor λ (see the appendix) equivalent to a 4-week effective memory length *n*_λ_,

(5) $$n_{\lambda }=\frac{\lambda }{1-\lambda }$$

which corresponds to λ = 0.9655 See the appendix for a description of the updating procedure. The RLS recursive solution, using finite impulse response (FIR) filters and described in detail in the appendix, is then used in the following algorithm: For a k-step ahead prediction of the background we compute multiple predictions for each day as follows. Denote the current step by *n*. Then using the available data we make the following predictions:

(6) $$\begin{array}{cc} \{y_{n-j-(k-1)},...,y_{n-j}\}\to {\hat{y}}_{n-j+k}, & j=0,...,k-1 \end{array}$$

Thus in our method each day is predicted multiple times. Among the multiplicity of the error terms to be fed back into the recursive algorithm update equations, we have obtained better background estimates by using the error with the smallest magnitude.

## Results and discussion

**Figure 7:**
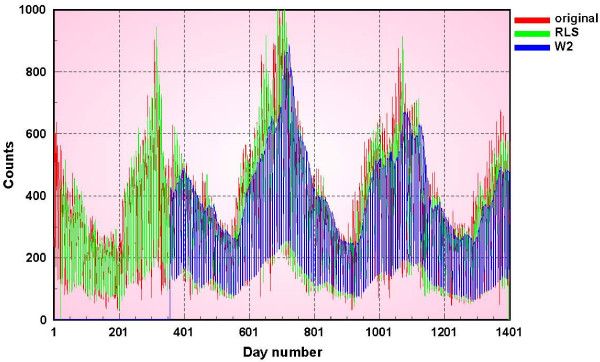
**Respiratory 1 data prediction results**.

We have applied the methods of the previous section to several univariate data streams. The first data set is respiratory 1 syndromic data (defined earlier) from military outpatient clinics of a major metropolitan area in the United States. In figure 7 the daily counts for a 3-year period 1994–1997 are shown in red. The green plot is the output of a 7-days ahead background prediction using our weekday-corrected, recursive least squares prediction method, using a 1 year training period for the day of the week correction. The blue plot is the result of the CDC prediction method W2 with a baseline of 4 weeks and a gap of 1 week. Although it appears that the RLS predictions have started much earlier than those of the W2 method, these predictions are based on using the first year of data to make the day of the week corrections and so we exclude them in making comparisons with other methods. Consequently we begin the W2 background predictions and show those values after 1 calendar year. The RLS predictions are consistently better than the W2 specially during the periods when the data are rising. When the time series are falling, the predictions are very close to each other.

**Figure 8:**
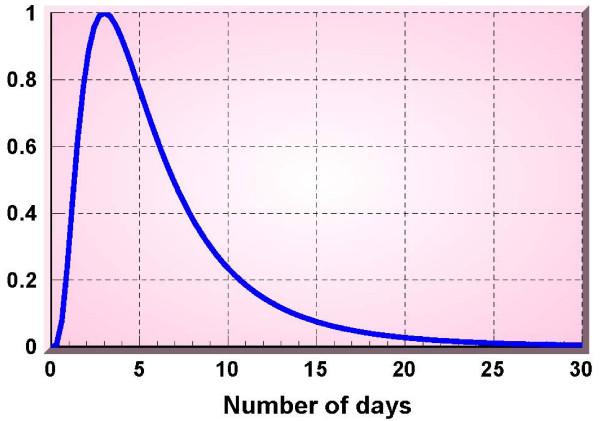
**Multiplicative signal**.

In order to better quantify the differences in the context of a biosurveillance alerting system, we performed an analysis of the detection performance of our method and compared them to one using the W2 predictions. This analysis was based on the injection of a log-normal signal into authentic time series of clinical visit counts. The signal type follows the observation of Sartwell [9] that the distribution of incubation periods of a point-source, common vehicle disease outbreak is approximately log-normal. The signal in this analysis was proportional to the deterministic form $e^{-{\left[\log(t/10)\right]}^{2}}$. This signal, depicted in figure 8, has a rise time of 3 days and a gradual fall time. The injection of the signal was multiplicative, with the resultant series computed as *y*(*t*){1 + α*s*(*t*)}. Our detector design is based on the multiplicative signal model. If the signal is present on day *t*, the daily count is *y*(*t*)(1 + α*s*), whereas when the signal is absent it is *y*(*t*). The test statistic is then the ratio of the two counts, which under ideal conditions is either 1 (signal absent) or 1 + α (signal present). The parameter α indicates the maximum signal level. Values of 0.25, 0.50, 0.75 and 1, corresponding to percent increases in counts of 25, 50, 75 and 100 were used.

We chose the Respiratory-1 data consisting of 1402 consecutive authentic daily visit counts as our background time series. For each given day, the estimated (predicted) background was used to normalize the actual daily count for that day (a simple division), and the result thresholded at different levels. Note that the day under consideration matches the maximum signal level, in the sense that if day *n *is being tested for the presence or absence of the signal, then the signal injection would have been such that

(7) data[*n *- *T*_*rise*_: *n *+ *T*_*Fall*_] = backgrond[*n *- *T*_*rise*_: *n *+ *T*_*Fall*_] × (1 + α*s*[*])

where *T*_*Rise *_and *T*_*Fall *_indicate the rise time and the fall time of the signal and the index *n *matches the index at which the signal *s*(*t*) is at its maximum value of 1.

A detection was recorded each time a threshold was exceeded. We found the probability of detection by dividing the total number of detections by the total number of available days, when the signal was actually present on the given day. Similarly, in the absence of the signal, a probability of false alarm was computed. The false alarm rate per number of days of interest was computed by multiplying the false alarm probability by the number of days of interest, e.g. the false alarm rate per week was found by multiplying the false alarm probability by 7.

**Figure 9:**
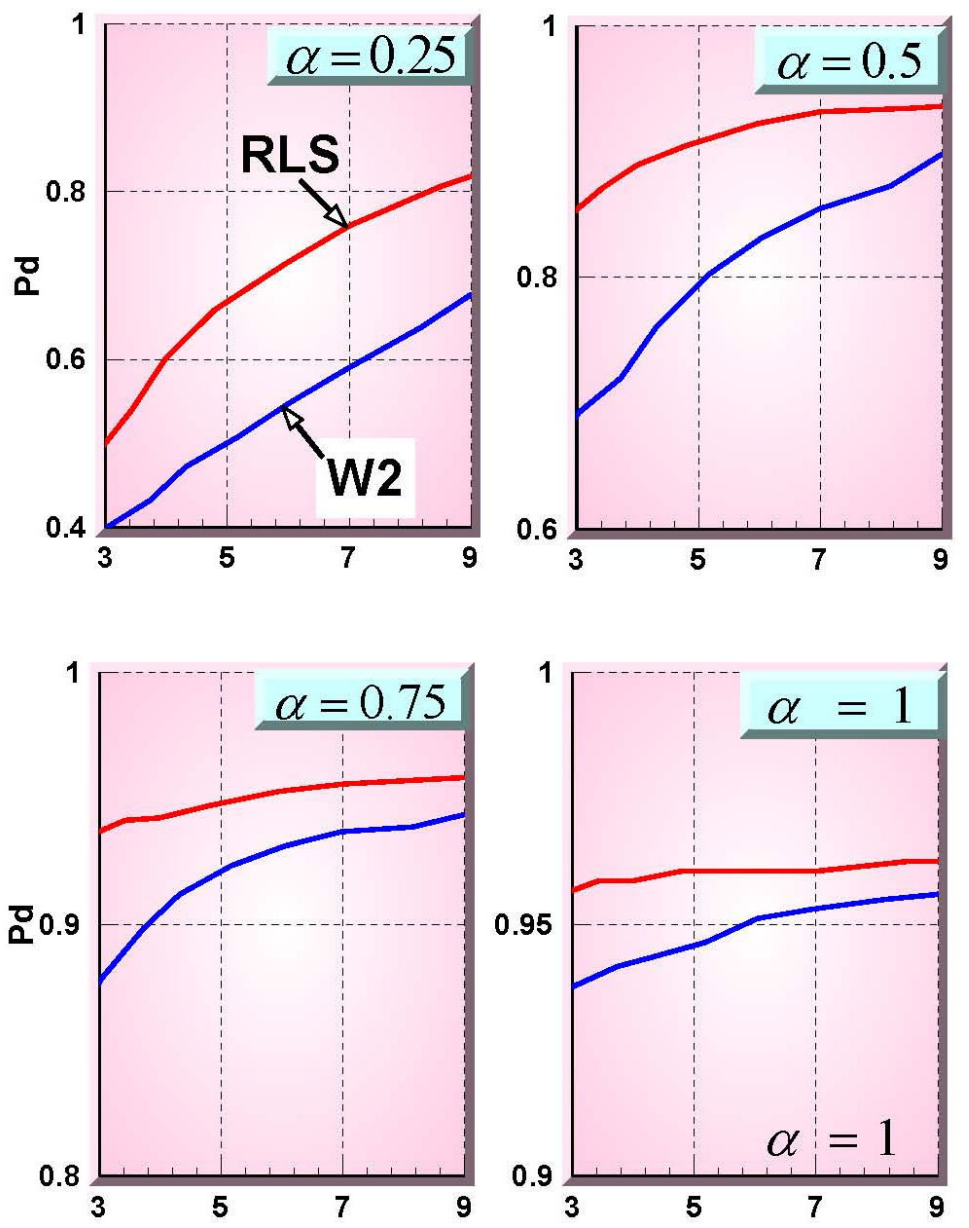
**Respiratory 1 – ROC curve – RLS (red) vs W2 (blue)**. The X-axis represents the number of false alarms every 28 days. Note that the Y-axis ranges are different for each plot.

Figure 9, shows 4 ROC (Receiver Operating Characteristic) curves [10] for the 4 different maximum signal values represented by the parameter α. The ROC curve relating to the RLS method is shown in red and that relating to the W2 method is shown in blue. Improvement at low false alarm probabilities are obtained with the RLS method described in this paper. The RLS results are exceptionally better than the baseline W2 method for the low signal maximum values (weak signals).

Next set of data consists of aggregated counts of syndromic surveillance data from the BioALIRT (Bio-Event Advanced Leading Indicator Recognition Technology) program that was conducted by DARPA [11]. This program was implemented during 2001–2004 with the objective to develop technologies and prototypes for monitoring nontraditional data sources (e.g., animal sentinels, human behavioural indicators, and non-diagnostic medical data) that might enable public health authorities to detect terrorist release of a pathogen or toxin.

**Figure 10:**
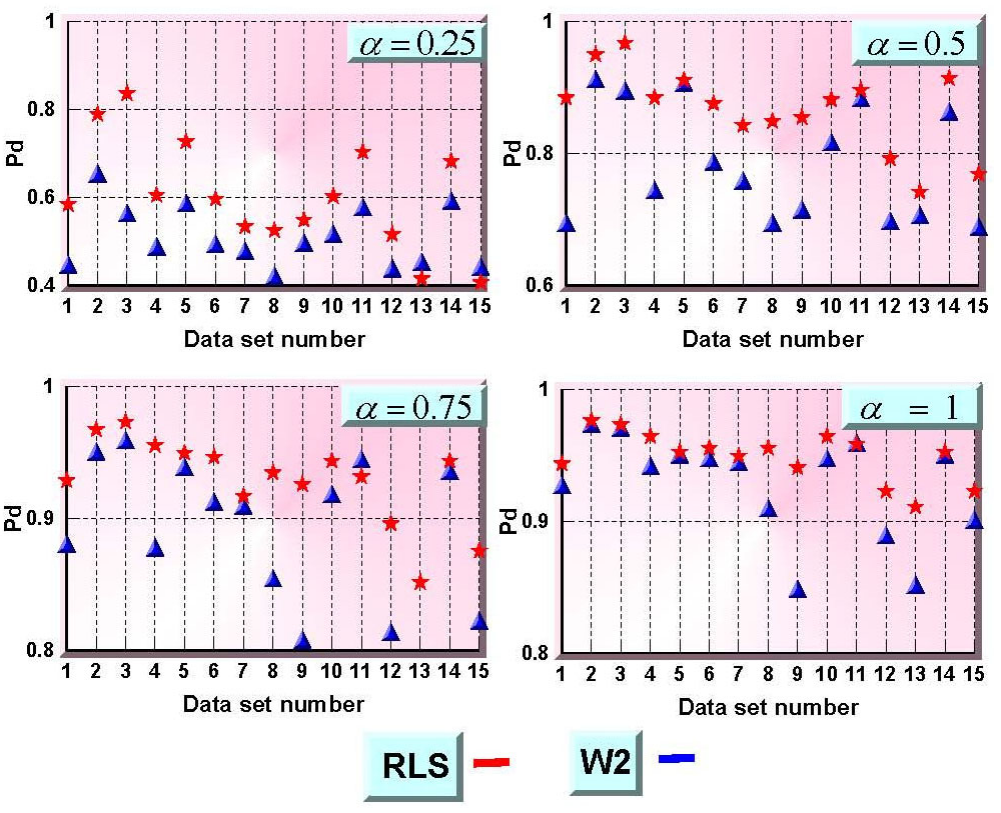
**Probabilities of detection for fixed false alarm rate of 1 per week, and α = 0.25, 0.5, 0.75 and 1, corresponding to maximum count increases of 25, 50, 75 and 100 percent – RLS (red) vs W2 (blue) – X-axis represents all 15 data sets (rows 2 through 16 of Table **1**)**. [Note that the Y-axes have different ranges.].

Data comprises three types of daily syndromic counts from ten large metropolitan areas: diagnoses from military clinics, filled military prescriptions, and civilian physician office visits. Out of the available 30 time series we selected 15 of them with the largest mean values, and they represent daily visit counts classified in Respiratory (Resp) and Gastrointestinal (GI) syndrome groups. These data together with their means and standard deviations are listed in Table 1. The first row of Table 1 lists the values for the Respiratory 1 data of the first data set; rows 2 through 16 are the remaining 15 time series from the BioALIRT program. Same signal injections were performed, and ROC curves were computed. Figure 10 shows the probabilities of detection for a fixed false alarm rate of one per week, for four different injection parameter values a. Better results, specially at the low values of a, over all 15 data sets are obtained using the RLS method.

## Conclusion

The current paper introduces a prediction approach for city-level biosurveillance data streams such as time series of outpatient clinic visits and over-the-counter remedy sales. This approach uses recursive-least-squares filters modified by a correction for the weekly patterns often seen in these data series. Unlike regression methods, these filters have the ability to adapt quickly to short-term trends, and this ability is essential for sensitivity to anomalies on a daily basis. Unlike some other adaptive methods based on autoregressive error modeling, this approach is applicable to many data types without detailed analysis. This flexibility is essential in surveillance applications.

For the study presented above, we formed a threshold detection algorithm from the residuals of these RLS forecasts. We compared the detection performance of this algorithm to the W2 method recently implemented at CDC. Like the day-of-week normalization above, the W2 method is a modification of the standard C2 aberration detector for improved handling of day-of-week effects. These 2 detectors were compared for power to detect realistic, simulated signals injected into each of a set of 16 actual datasets. These datasets were drawn from several sources, and the modified RLS-based method gave consistently better sensitivity at multiple background alert rates. Thus, this forecast technique should be further considered for routine application in biosurveillance systems.

Limitations of this modified RLS forecast method are the scale of time series required for application of the weekly pattern correction and the amount of data history required for useful filter coefficients. Regarding the necessary data scale, this forecaster gave improvements for time series with mean values as low as 30 counts per day, but informal tests have suggested that the method is applicable for lower scales as long as weekend counts do not drop off to zero. For the training data issue, 25% of the 700 days of the BioALIRT data-about a 6-month warmup-was used for the RLS forecasts in the simulation comparisons. The effective extent of these limitations and possible enhancements to overcome them are subjects for further research.

The RLS adaptation presented here should also be considered for multivariate forecasts. The adaptive modeling of cross-correlation effects combined with the ability to capture trends on a short time scale suggest a possible detection advantage over multivariate statistical process control charts. The increasing availability of multiple data sources emphasizes the need for tools that can effectively combine various types of statistical evidence.

## Abbreviations

OTC: over the counter (medications); BioALIRT: Bio-Event Advanced Leading Indicator Recognition Technology; RLS: Recursive Least Squares.

## Competing interests

The authors declare that they have no competing interests.

## Authors' contributions

The idea of predicting clinical data using recursive least squares with feedback of minimum error among multiple looks, and the associated computer programs were developed by AHN. Data was provided by HB, who also wrote most of the conclusion, and contributed to the responses to the referees.

## Pre-publication history

The pre-publication history for this paper can be accessed here:

http://www.biomedcentral.com/1472-6947/9/4/prepub

## Supplementary Material

Additional file 1 **Recursive least squares prediction.** Mathematical description of the algorithm for background prediction presented in this paper. Click here for file
