# Supplementary material for: Recursive least squares background prediction of univariate syndromic surveillance data
Source: BMC Med Inform Decis Mak. 2009 Jan 16;9:4. doi: 10.1186/1472-6947-9-4 (PMC2639573; doi:10.1186/1472-6947-9-4)
Supplement: Additional file 1 — Recursive least squares prediction. Mathematical description of the algorithm for background prediction presented in this paper. [file 1472-6947-9-4-S1.pdf]

Additional file 1

Recursive least squares prediction

The problem of predicting a non-stationary time series y_n using a single correlated reference channel represented by a time series x_n is described here [1]. We limit this appendix to one reference channel without loss of generality. For the purposes of univariate data we simply choose $x_n = y_n$.

Let $\hat{y}_{n+k}$ denote the predicted time series when data at time steps $\leq n$ are available, i.e. we are making a prediction k steps ahead by using the linear prediction equation $\hat{y}_{n+k} = \sum_{m=0}^{M-1} h_m x_{n-m}$. We define a performance index $\varepsilon_n = \sum_{l=0}^{n} \lambda^{n-l} |e_l|^2$ where the error at each step is defined by $e_l = y_l - \hat{y}_l$, and λ is the forgetting factor introduced to enforce adaptation of the filter to the most recent changes in the data. The role of the forgetting factor can be understood by considering the quantity $n_\lambda = \left(\sum_{n=0}^{\infty} \lambda^n\right)^{-1} \left(\sum_{n=0}^{\infty} n\lambda^n\right) = \lambda(1-\lambda)^{-1}$ to be a measure of the effective memory of the filter. Smaller values for λ correspond to shorter memory lengths n_λ enabling better tracking of nonstationarities by the filter. Defining the matrix elements $R_{ij}[n] = \sum_{l=0}^{n} \lambda^{n-l} x_{k-i} x_{k-j}$ and the vector elements $r_i[n] = \sum_{l=0}^{n} \lambda^{n-l} y_k x_{k-i}$, the Normal equations at any step are $\mathbf{R}[n]\,\mathbf{h}^{(n)} = \mathbf{r}[n]$, showing the dependence of the filter on each step n. The quantities $\mathbf{R}$ and $\mathbf{r}$ satisfy rank-one update equations, namely, $\mathbf{R}[n] = \lambda\mathbf{R}[n-1] + \mathbf{x}[n]\,\mathbf{x}^T[n]$, and $\mathbf{r}[n] = \lambda\mathbf{r}[n-1] + \mathbf{x}[n]\,y_n$. Standard rank-one update techniques then lead to the following recursive least squares algorithm (if we denote apriori quantities by a subscript 0, and apostiori quantities by a subscript 1):

$$\mathbf{P}_0 = \mathbf{R}_0^{-1}, \;\; \mathbf{K}_0 = \mathbf{P}_0\mathbf{x},$$

$$\nu = \mathbf{K}_0^T\mathbf{x}, \;\; \mu = (1+\nu)^{-1},$$

$$\mathbf{K}_1 = \mu\mathbf{K}_0, \;\; \mathbf{P}_1 = \mathbf{P}_0 - \mathbf{K}_1\mathbf{K}_0^T,$$

$$\hat{y}_0 = \mathbf{h}_0^T\mathbf{x}, \;\; e_0 = y - \hat{y}_0,$$

$$\mathbf{h}_1 = \mathbf{h}_0 + e_0\mathbf{K}_1$$

The recursion begins with a zero filter and a covariance matrix that is a large multiple of the unity matrix. The multiple look feature introduced in this paper relates to the prediction error that is fed back into the filter update (the last step).

This error term is computed multiple times for any given day index using multiple filters depending on the number of steps for the prediction.

References

[1] Moon TK, Stirling WC: *Mathematical Methods and Algorithms for Signal processing*. New Jersey: Prentice-Hall; 2000
